# First‐line treatment of stage IIB to stage IV classical Hodgkin lymphoma in Italy, Israel, and Spain: Patient characteristics, treatment patterns, and clinical outcomes

**DOI:** 10.1002/jha2.426

**Published:** 2022-04-19

**Authors:** Abraham Avigdor, Fabrizio Trinchese, Francois Gavini, Nawal Bent‐Ennakhil, Mehul Dalal, Athanasios Zomas, Sharmeen Gettner Broun, Guido Gini

**Affiliations:** ^1^ The Chaim Sheba Medical Center Institute of Hematology Ramat Gan Israel; ^2^ Sackler Faculty of Medicine Tel Aviv University Tel Aviv Israel; ^3^ Takeda Pharmaceuticals International AG Zurich Switzerland; ^4^ Millennium Pharmaceuticals, Inc. A wholly owned subsidiary of Takeda Pharmaceutical Company Ltd Cambridge Massachusetts USA; ^5^ Kantar Paris France; ^6^ Ospedali Riuniti di Ancona Clinic of Hematology, Ancona, Italy

**Keywords:** Advanced stage Hodgkin's lymphoma, clinical outcome, frontline chemotherapy, interim PET scans, safety

## Abstract

Classical Hodgkin lymphoma (cHL) is curable in 90% of cases, but advanced stage patients who do not respond well to first‐line (1L) therapy have poorer outcomes. This retrospective study examines patient characteristics, treatment patterns, clinical outcomes, and safety management of 1L cHL therapies in common clinical practice in Italy (IT), Israel (IL), and Spain (SP). The overall sample (*n* = 256) included patients with stage IIb to IV cHL, of which 86.3% received ABVD as 1L therapy (*n* = 221). Clinical outcomes were similar for the overall population and ABVD subsample: complete response (CR) in 75% and 76.5%; 30‐month (30‐mo) survival (OS) of 92.5% and 93.6%; and 30‐mo progression‐free survival (PFS) of 70.7% and 72.6%. Thirty‐month PFS was significantly lower for patients ≥ 60 years and/or with high (4–7) IPS. Treatment‐induced pulmonary and cardiac toxicities, and febrile neutropenia occurred, respectively, in 10%, 2.3%, and 6.8% of ABVD‐treated patients. Interim PET or PET‐CT scans were performed after two cycles of 1L therapy (PET2) for 70.3% and 66.6% of the overall and ABVD cohorts, respectively. PET2 positive rates were nearly 30% (49/173), yet PET‐adapted strategy of dose modification only occurred in a small fraction of patients.

## 1 INTRODUCTION

Classical Hodgkin lymphoma (cHL) is a highly curable malignancy with a reported 90% survival at 5 years [1]. However, patients with advanced stage cHL who fail to respond to front‐line therapy generally fare a poor prognosis. Outcomes for patients with relapsed or refractory cHL have greatly improved in the era of immunomodulatory and antibody‐drug conjugate therapies, but toxicity associated with long‐term treatment represents a considerable burden in these patients [2].

In the last two decades, the standard‐of‐care for patients with previously untreated advanced stage cHL has included two chemotherapy regimens, administered either alone or followed by radiation therapy (RT) [3, 4]. These are doxorubicin, bleomycin, vinblastine and dacarbazine (ABVD)‐ or a variant minus bleomycin (AVD)‐ and dose‐escalated bleomycin, etoposide, doxorubicin, cyclophosphamide, vincristine, procarbazine and prednisone (eBEACOPP) [5, 6, 7]. Although highly effective, these treatments are associated with a significant risk of adverse events (AEs) and toxicity, in particular bleomycin‐related pulmonary toxicity, cardiotoxicity, neutropenia, and other complications such as infection, anemia, peripheral neuropathy, and alopecia [8, 9]. Treatment with eBEACOPP may result in a marginally improved overall survival (OS) at 5 years [10, 11], but is more toxic than ABVD [12, 13]. Recently, a phase III clinical trial pointed out brentuximab vedotin (A) as a promising alternative to bleomycin, in combination with AVD, for 1L treatment [14].

Physician treatment choice is currently made according to risk stratification provided by the International Prognostic Score (IPS), age, disease stage and, more recently is being adjusted by interim response assessment commonly provided by positron emission tomography (PET) and/or computed tomography (CT) scans [15, 16]. Results from PET/CT performed after 2 cycles of therapy (PET2) are used for response‐adapted therapy management, offering a balance between risk of AE and survival outcomes [17]. When PET2 findings are deemed negative, the most commonly adopted approach is to discontinue the compound with the weakest anti‐lymphoma activity, bleomycin, in order to reduce the risk of pulmonary toxicity [18] or to reduce the number of treatment cycles [19]). Conversely, the treatment intensity of PET2‐positive patients may be increased, most often by switching to eBEACOPP, but at the cost of an increased rate of acute toxicity [20], gonadal damage, and second malignancies [21]. Nonetheless, PET2‐driven strategies have certain limitations. PET scans are not always easily interpretable and PET2‐negative patients do not always display long‐term remissions [21, 22].

Evidence from clinical practice allows to review the effectiveness and safety of cHL treatments in cohorts representative of the general population, including patients with comorbidities who may not be included in clinical trials. The aim of this study was to examine patient characteristics, treatment patterns, safety management, and clinical outcomes associated with 1L systemic regimens used to treat advanced stage cHL in Israel (IL), Italy (IT), and Spain (SP).

## 2 MATERIALS AND METHODS

### 2.1 Study design

This is an observational study consisting of a multicentric retrospective chart review analysis of 256 cHL patients, selected by hematologists and oncologists across large academic reference centers in IL (*n* = 1), IT (*n* = 13), and SP (*n* = 14). Hmatologists/oncologists were randomly selected, while ensuring some level of geographic distribution across the countries and across settings (hospital [teaching/non‐teaching], cancer care unit or clinic), to ensure a case‐mix of different practice settings and protocols. Adult patients (18 years old or over) who signed an informed consent; initially diagnosed between March 2014 and August 2018 with Ann Arbor Clinical disease stage IIb (with extra‐nodal involvement and/or large or bulky mediastinal tumor), III or IV cHL; receiving systemic chemotherapy as 1L treatment, with or without RT were eligible for study inclusion. Inclusion of patients with stage IIb disease was in line with German Hodgkin Disease Study Group (GHDSG) classification and clinical trial populations [6, 20]. Patients who received 1L therapy as part of a clinical trial were excluded. The decision to perform interim PET2 scans was based on clinical judgment of treating physicians. Physicians were asked to submit complete patient treatment details and survival outcomes, and indicate AEs and toxicities based on a list of common events, from the time of initial cHL diagnosis to the most recent follow‐up visit, in electronic case report forms.

### 2.2 Statistical analyses

Descriptive statistics examined patient characteristics, 1L treatment modalities, AE and clinical outcomes, by age (< or ≥ 60 years old), IPS, disease stage, and extra‐nodal involvement. Patient characteristics are presented as median (range) and mean (standard deviation, SD) for continuous variables and number and percentages for categorical variables. Missing data were not imputed for analyses.

The 1L systemic treatment groups were ABVD, AVD, eBEACOPP including de‐escalated to ABVD (eBEACOPP_initiation‐based_), or other systemic therapy.

OS, PFS, and complete response (CR) at the end of 1L therapy were described. OS was defined as the time between the start date of the 1L systemic therapy (index date) and the date of death (regardless of the cause). PFS was defined as the time from the index date to the time when disease progression or death were first observed. OS and PFS were estimated using Kaplan–Meier (K‐M) survival analysis methods. *P*‐values and two‐sided 95% confidence interval (CI) of estimates were compared using Log rank test.

Version 9.4 of SAS® software (SAS Institute, Cary, NC, USA) was used for all statistical analyses.

## 3 RESULTS

### 3.1 Patient characteristics

The demographic and baseline disease characteristics of the study population by systemic 1L therapies are listed in Table 1. The overall cohort comprised 256 patients initially diagnosed with advanced stage cHL, of which 51.2% were diagnosed with stage IV, 34.3% with stage III, and 14.5% with stage IIb cHL (with bulky or extra‐nodal disease). Thirty‐five percent of patients were diagnosed after 2016. The median age at cHL diagnosis was 39 years (range: 19–91 years). Overall, 42 patients (16.4%) were aged ≥ 60 years, with slightly more males (54.3%) affected (sex ratio 1.19). Patients were distributed across SP (41.3%), IT (35.5%), and IL (23.2%), with a moderately higher number of older (≥60 years) patients included in SP (21.5%) versus IT (15.7%) and IL (8.3%). All other patient characteristics were largely consistent across the three countries.

**TABLE 1:** Demographic and baseline disease characteristics of the overall study population by first‐line (1L) systemic treatment. Data are listed by country (IL, IT, and SP) or by age (< and ≥ 60 years old). CCI, Charlson comorbidity index; ECOG, Eastern Cooperative Oncology Group; IL, Israel; IT, Italy; SP, Spain

		**ABVD (*N* = 221)**	**AVD (*N* = 7)**	**BEACOPP** **^†^** **(*N* = 22)**	**Other (*N* = 6)**	**Overall total (*N* = 256)**
		***N* (%)**	***N* (%)**	***N* (%)**	***N* (%)**	***N* (%)**
**Age**	Mean (SD)	42.2 (16)	66.7 (17.3)	41.2 (10.6)	78 (8.0)	40.7 (16.9)
	Median [range]	39 [19–83]	73 [35–82]	38 [21–55]	78 [65 –90]	39 [19–91]
**Gender**	Male	118 (53.4%)	4 (57.1%)	15 (68.2%)	2 (33.3%)	139 (54.3%)
**Disease stage at initiation of 1L treatment**	IIb‐III	113 (51.1%)	4 (57.1%)	6 (27.3%)	0	123 (48.0%)
	IV	108 (48.9%)	3 (42.9%)	16 (72.7%)	6 (100.0%)	133 (52.0%)
	Non bulky (< 10 cm)	147 (66.5%)	6 (85.7%)	15 (68.2%)	5 (83.3%)	173 (67.6%)
	Bulky (≥ 10 cm)	49 (22.2%)	0	7 (31.8%)	0	56 (21.9%)
	Unknown	25 (11.3%)	1 (14.3%)	0	1 (16.7%)	27 (10.5%)
**IPS at diagnosis**	Low intermediate: [0–3]	144 (65.2%)	2 (28.6%)	13 (59.1%)	2 (33.3%)	161 (62.9%)
	High: [4–7]	34 (15.4%)	4 (57.1%)	9 (40.9%)	2 (33.3%)	49 (19.1%)
	Unknown	43 (19.5%)	1 (14.3%)	0	2 (33.3%)	46 (18.0%)
**Extranodal disease at initiation**	Yes	127 (57.5%)	3 (42.9%)	18 (81.8%)	5 (83.3%)	153 (59.8%)
	No	92 (41.6%)	4 (57.1%)	4 (18.2%)	1 (16.7%)	101 (39.5%)
	Unknown	2 (0.9%)	0	0	0	2 (0.8%)
**Patient performance status at diagnosis**	ECOG = 0–1	129 (58.4%)	6 (85.7%)	17 (77.3%)	4 (66.7%)	156 (60.9%)
	ECOG = 2+	29 (13.1%)	0	0	0	29 (11.3%)
	Unknown	63 (28.5%)	1 (14.3%)	5 (22.7%)	1 (16.7%)	70 (27.3%)
**CCI** **ˆ** **at 1L initiation**	Median index (range)	2.0 [2–11]	5 [2–9]	2.0 [2–4]	6.5 [5–8]	2.0 [2–11]
	Mean index (SD)	3.0 (1.8)	5.5 (2.2)	2.4 (0.7)	6.5 (1.0)	3.1 (1.8)
**B‐symptoms**	At initiation of 1L	114 (51.6%)	4 (57.1%)	18 (81.8%)	3 (50.0%)	139 (54.3%)
	Fatigue	67 (30.3%)	3 (42.9%)	11 (50.0%)	3 (50.0%)	84 (32.8%)

Abbreviations: ABVD, doxorubicin, bleomycin, vinblastine and dacarbazine; AVD: a variant of ABVD minus bleomycin.

^†^ BEACOPP_initiation based_, bleomycin, etoposide, doxorubicin, cyclophosphamide, vincristine, procarbazine and prednisone; ˆCCI, Charlson comorbidity index (index range includes +2 for lymphoma); 1L, first line; B‐symptoms, (i.e., weight loss, fever and/or night sweats); SD, standard deviation.

Overall, 62.9% patients included in this study had low to intermediate IPS (0‐3) at diagnosis and 59.8% presented with extra‐nodal disease. Forty‐six (46) of the 256 patients (18.0%) had at least one comorbidity at the time of treatment initiation, of whom 19 patients (45.2%) were aged ≥ 60 years. The most frequently reported comorbidities were chronic pulmonary disease (CPD, 5.1%, *n* = 13) and diabetes (4.3%, *n* = 11), and AIDS/HIV (3.1%, *n* = 8). The prevalence of these comorbidities was similar in all patients, with the exception of HIV/AIDS which was reported mostly in younger patients (*n* = 7 in < 60 years vs. *n* = 1 ≥ 60 years). The mean Charlson comorbidity index (CCI) at cHL diagnosis was 4.5 (median: 3.0, range: 3.0–9.0) and was slightly higher in younger patients (*n* = 27 < 60 years with mean CCI 4.9). The number of patients with one or more comorbidities was slightly higher in SP (25.2%; *n* = 27) than IT (13.5%; *n* = 12) or IL (11.7%, *n* = 7). This may be related to the slightly older age and/or the inclusion of patients with HIV/AIDS reported only in SP.

Over half (54.3%, *n* = 139) of patients experienced one or more cHL‐related B symptoms (i.e., weight loss, fever, and/or night sweats) and 32.8% (*n* = 84) complained of fatigue at the time of treatment initiation. Eastern Cooperative Oncology Group (ECOG) performance status was 0–1 for 60.9% (*n* = 156) of all patients.

### 3.2 Treatment modalities

The 1L treatments are shown in Table 2. The predominant 1L treatment was ABVD (86.3%, *n* = 221), then eBEACOPP_initiation‐based_ (8.6%, *n* = 22), AVD (2.7%, *n* = 7), and other systemic non‐documented therapy (2.3%, *n* = 6), for a mean duration of 22.2 (±4.2) weeks. Of note, stage IV cHL patients represented 48.9% of ABVD‐ and 42.9% of AVD‐treated patients, versus 72.7% of eBEACOPP_initiation‐based_ ‐treated patients.

**TABLE 2:** First‐line treatment, including type of chemotherapy regimen, interim PET2‐scans and radiation therapy (RT). ABVD, doxorubicin, bleomycin, vinblastine and dacarbazine; AVD, doxorubicin, vinblastine and dacarbazine; eBEACOPP_initiation‐based doxorubicin_, cyclophosphamide, etoposide, vincristine, bleomycin, procarbazine and prednisone

		**ABVD (*N* = 221)**	**AVD (*N* = 7)**	**BEACOPP** **^†^** **(*N* = 22)**	**Other (*N* = 6)**	**Overall total (*N* = 256)**
		***N* (%)**	***N* (%)**	***N* (%)**	***N* (%)**	***N* (%)**
**Duration of 1L therapy (weeks)**	*N*	191	7	22	5	225
	Mean (SD)	22.5 (4.2)	22.3 (2.3)	19.6 (3.5)	19.2 (4.9)	22.2 (4.2)
	Median [range]	22.4 [6.0–45.0]	22.0 [18.6–26.3]	22.1 [10.3–26.0]	19.1 [14.4–25.9]	22.3 [6.0–45.0]
**Cycles completed**	Less than 6 cycles	18 (8.1%)	0	0	1 (16.7%)	36 (14.1%)
	6 Cycles	187 (84.6%)	6 (85.7%)	19 (86.4%)	3 (50.0%)	215 (84.0%)
	8 Cycles	5 (2.3%)	0	0	0	5 (2%)
**Interim PET2** **^‡^** **evaluations**	Yes	142 (66.6%)	7 (100.0%)	20 (95.3%)	4 (80.0%)	173 (70.3%)
	No/unknown	79 (35.7%)	0	2 (9.1%)	2 (33.3%)	83 (32.4%)
	*N*	142	7	20	4	173
**PET2** **^‡^** **evaluation results**	Positive	42 (29.6%)	1 (14.3%)	4 (20.0%)	2 (50.0%)	49 (28.3%)
	Negative	96 (67.6%)	6 (85.7%)	16 (80.0%)	2 (50.0%)	120 (69.4%)
	*N*	142	7	20	4	173
**Dose modifications**	Dose escalation	15 (10.5%)	0	0	0	15 (8.6%)
	Dose de‐escalation	12 (8.5%)	0	15 (75%)	1 (25%)	28 (16.2%)
	no change	109 (76.8%)	7 (100%)	5 (25%)	2 (50%)	123 (48%)
	Undocumented	6 (4.2%)	0	0	1 (25%)	7 (4%)
**Radiation therapy (RT)**	*N*	32	0	0	1	33
	Mean (SD)	5.9 (9.1)				5.8 (9.0)
**Site of radiation treatment**	*N*	32	0	0	1	33
	Localized	23 (71.9%)			1	24 (72.7%)
	Regional	8 (25.0%)				8 (24.2%)
	Extensive	1 (3.1%)				1 (3.0%)
**Rational for RT**	*N*	32			1	33
	Consolidative therapy^§^	21 (65.6%)			1	22 (66.7%)
	Residual disease	10 (31.3%)				10 (30.3%)

Abbreviations: ABVD: doxorubicin, bleomycin, vinblastine and dacarbazine; AVD: a variant of ABVD minus bleomycin.

^†^ BEACOPP_initiation based_, bleomycin, etoposide, doxorubicin, cyclophosphamide, vincristine, procarbazine and prednisone; PET2, Positron Emission Tomography at cycle 2.

^‡^ PET/PET‐CT scan; ; SD: standard deviation.

^§^ Therapy without residual disease.

Most patients (84.0%) completed 6 cycles of 1L therapy, while 14.1% interrupted treatment earlier. Among the 29 patients who ceased 1L chemotherapy, 10 stopped because of disease progression, 4 because of AEs (toxicity and/or infection), and 2 died before completing treatment. In the 1L ABVD cohort, 187 (84.6%) patients completed 6 cycles of therapy and 5 patients (2.3%) completed 8 cycles. Of note, 54 patients (24.4%) of the ABVD cohort received second line (2L) systemic therapy, alone or in combination with ASCT for 18 patients (8.1%, data not shown).

The median age of the study population was 39 years and similar in both ABVD and eBEACOPP_initiation‐based_ treatment cohorts. Whereas most of the 42 patients aged ≥ 60 years were treated with ABVD (73.8%; *n* = 31), 5 (11.9%) were preferentially given AVD, the exclusion of bleomycin allowing a better tolerance of chemotherapy. Conversely, only younger (median age 38 [range 21–55] years), patients were treated with the more aggressive combination eBEACOPP_initiation‐based_.

A total of 173 (70.3%) of the study population (*n* = 142, 66.6% in ABVD; *n* = 7, 100% in AVD; *n* = 20, 95.3% in eBEACOPP_initiation‐based_; *n* = 4, 80% in other) underwent PET2 scans to assess treatment efficacy. PET2 assessments were most often performed for more advanced stages of the disease, with over half (52.1%) of ABVD‐treated patients undergoing PET2 initially diagnosed with stage IV cHL (vs. 31.0% for stage III and 16.9% for stage II cHL). Among ABVD patients, 27 (19%) had PET2‐driven modifications. PET2 scans were positive in 29.6% (*n* = 42). Thirteen of the PET2‐positive patients (30.9%) were offered escalation from ABVD. PET2 results were negative in the other 96 patients (67.6%), of whom 12 (12.5%) had a de‐escalation and two (2), (2.1%) had escalation of treatment dose. Of note, PET2 scans were done with similar frequencies within the three countries (data not shown).

RT was undertaken by 12.9% (*n* = 33) of the overall population, nearly all of them (*n* = 32) following 1L ABVD chemotherapy. The anatomical site of radiation was localized (72%), regional (25%), or extensive (3%). RT was mostly given as a consolidation therapy in 65.6% (*n* = 21) of ABVD patients, or to treat residual disease in 31.3% (*n* = 10). Utilization of RT appeared to vary within countries, with only 3.3% (*n* = 2) of advanced stage cHL patients receiving RT in IL, against 19.1% (*n* = 17) in IT and 13.1% (*n* = 14) in SP.

### 3.3 Clinical outcomes

The mean (±SD) follow‐up duration was 31.0 (±14.2) months (median: 30.3 months, 95% CI, 2.4– 60.6 months) for the overall population and 31.2 (±14.1) months (median: 30.6 months, 95% CI, 2.4–60.6 months) for the 1L ABVD cohort.

**FIGURE 1:**
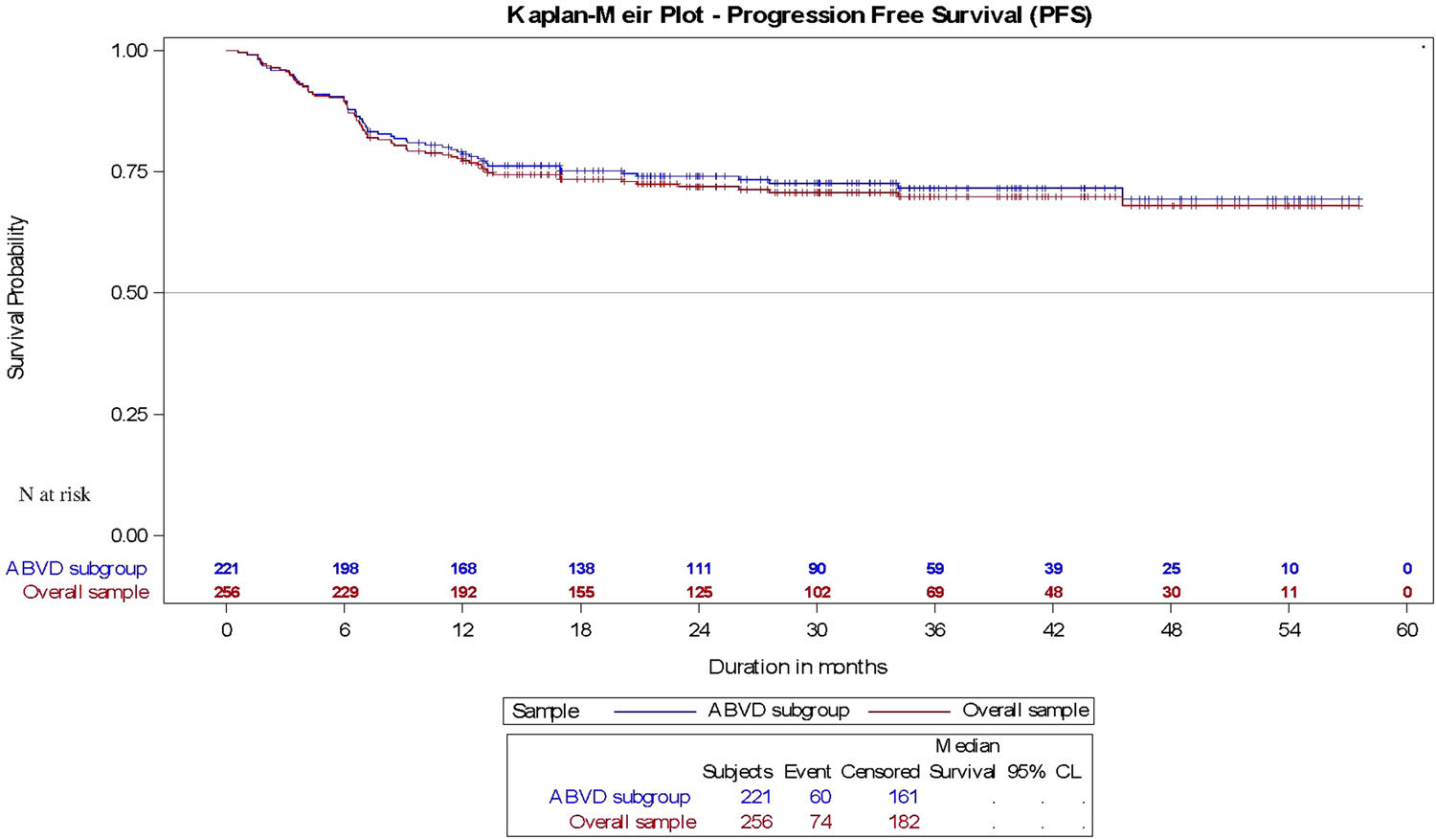
Kaplan–Meier curves for progression‐free survival (PFS) of the overall study population and the doxorubicin, bleomycin, vinblastine and dacarbazine (ABVD)‐treated subgroup; *N*, number of patients at risk

At the end of 1L treatment, CR was achieved for 75.0% of the overall study population and 76.5% of the 1L ABVD‐treated patients. The estimated 30‐mo OS was 92.5% (95% CI, 89.0%–96.0%) and 93.6% (95% CI, 90.1%–97.0%) for the overall and 1L ABVD cohorts, respectively (data not shown). A total of 60 patients (27.1%) of the 1L ABVD cohort experienced disease progression or died after or during treatment. The estimated 30‐mo PFS was 70.7% (95% CI, 66.6%–78.7%) for the overall, and 72.6% (95% CI, 69.3%–81.2%) for ABVD cohorts (Figure 1).

**FIGURE 2:**
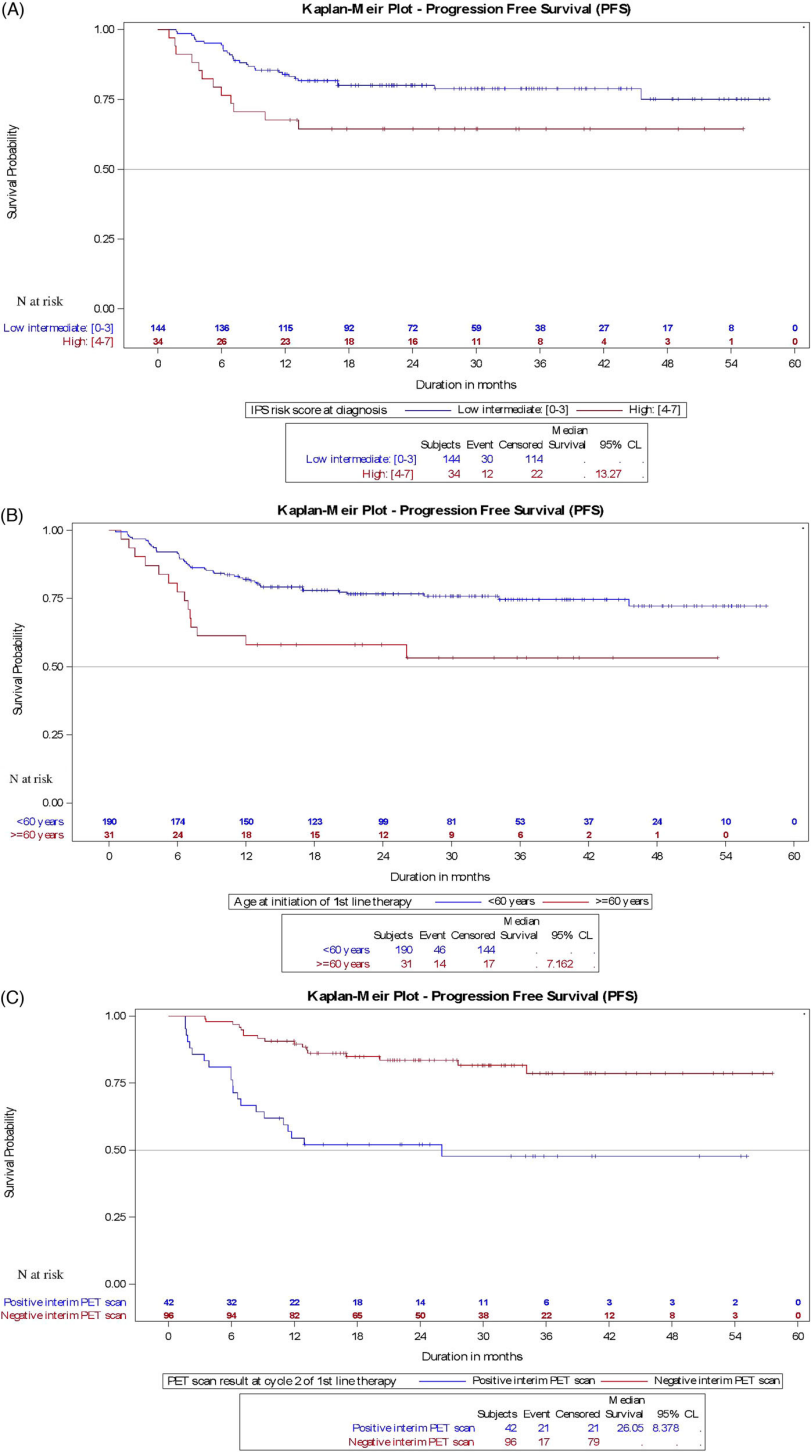
(A) Kaplan–Meier curves for progression‐free survival (PFS) of doxorubicin, bleomycin, vinblastine and dacarbazine (ABVD)‐treated patients by IPS (high [4, 5, 6, 7] vs. low [0–3]; IPS, international prognostic score; *N*, number of patients at risk. (B) Kaplan–Meier curves for PFS of ABVD‐treated patients by age (< 60 vs. > 60); *N*, number of patients at risk. (C) Kaplan–Meier curves for PFS of ABVD‐treated patients by PET2 results (PET2 positive vs. PET2 negative). PET2, positron emission tomography at cycle 2; *N*, number of patients at risk

**FIGURE 3:**
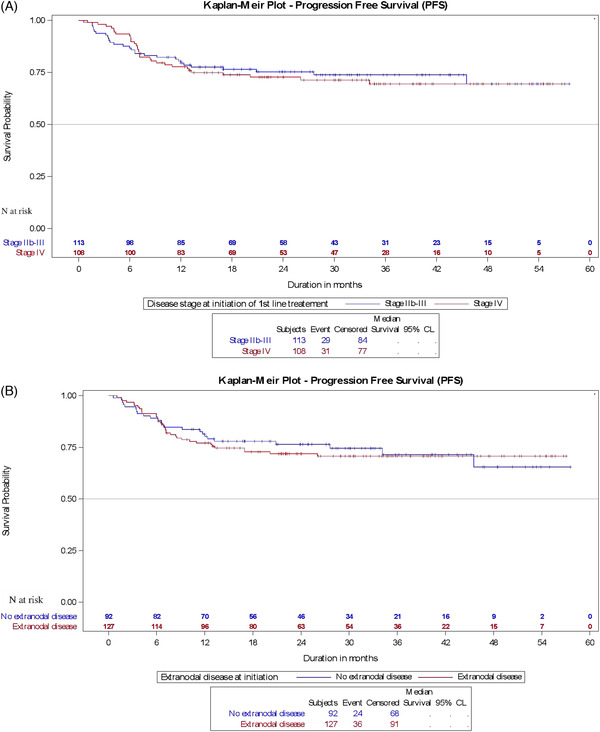
(A) Kaplan–Meier curves for progression‐free survival (PFS) of doxorubicin, bleomycin, vinblastine and dacarbazine (ABVD)‐treated patients by disease stage. *N*, number of patients at risk. (B) Kaplan–Meier curves for PFS of ABVD‐treated patients by the presence or absence of extranodal sites; *N*, number of patients at risk

As expected, these values were significantly lower for patients with high IPS (4‐7) compared to those with low to intermediate scores at diagnosis, both in the overall study sample (62% vs. 78%; *P *= 0.04) and in the 1L ABVD subgroup (64% vs. 79%; *P *= 0.02, Figure 2A). Likewise, the estimated 30‐mo PFS was significantly reduced for patients aged 60 or more, with only 47.6% (vs. 75.3% < 60 years; *P *= 0.0005) of the overall study population and 53.2% (vs. 75.8% < 60 years, *P *= 0.006) of the ABVD‐treated patients remaining progression‐free 30 months after 1L treatment (Figure 2B). As one would expect, the 30‐month PFS was also reduced for patients presenting with positive results at PET2 (Figure 2C). Although not statistically different, 30‐mo PFS was slightly lower for patients diagnosed with stage IV versus stage IIb–III disease, and for patients presenting with extra‐nodal disease, both in the overall population or the ABVD subgroup (Figure 3A,B). During these 30 months, 25.8% of the overall population and 24.4% of the ABVD cohort underwent subsequent 2L systemic chemotherapy, with 50.9% of the ABVD subgroup initially diagnosed with stage IV cHL receiving 2L treatment.

### 3.4 Safety and patient management

Overall, 31 patients interrupted 1L treatment, 26 of whom belonging to the ABVD cohort. The reasons for interruption of ABVD included pulmonary toxicity (*n* = 3), infection (*n* = 2), neutropenia (*n* = 1), and febrile neutropenia (*n* = 1). Moreover, ABVD dose was reduced for six patients suffering from pulmonary toxicity and one patient with neutropenia.

A total of 216 (84.4%) patients experienced at least one AE (Table 3). The most frequent AE was all grade neutropenia: in 48.8% (*n* = 125) and 47.1% (*n* = 104) of the overall and 1L ABVD cohorts, respectively, with a rate of febrile neutropenia of 10.9% (*n* = 28) and 6.8% (*n* = 15) in overall and ABVD cohorts. The second and third most frequent AEs were alopecia and anemia which affected, respectively, 31.2% (*n* = 69) and 27.6% (*n* = 61) of ABVD‐treated patients. Infections were reported for 22.2% (*n* = 49) patients in the ABVD subgroup. Pulmonary toxicity was reported in 10% (*n* = 22) and neuropathy in 5.9% (*n* = 13) of ABVD‐treated patients. Finally, cardiovascular and hepatic toxicity occurred, respectively in 5 (2.3%) and 6 (2.7%) ABVD‐treated patients. Of note, among the ABVD patients suffering from cardiovascular and/or pulmonary complications, 11 had undergone PET2 assessment and 3 had treatment de‐escalation.

**TABLE 3:** Treatment‐emergent adverse events (AEs) in the overall study population and the ABVD subgroup. In each cohort, AEs are presented by age (< and ≥ 60 years old). *Chi‐square test, Fisher exact *P* < 0.05

	**Systemic treatment 1L**											
	**ABVD (*N* = 221)**						**Overall total (*N* = 256)**					
			**Age < 60**		**Age ≥ 60**				**Age < 60**		**Age ≥ 60**	
	***N* = 221**		***N* = 190**		***N* = 31**		***N* = 256**		***N* = 214**		***N* = 42**	
	***N***	**(%)**	***N***	**(%)**	***N***	**(%)**	***N***	**(%)**	***N***	**(%)**	***N***	**(%)**
Neutropenia (all‐grade)	104	47.1	87	45.8	17	54.8	125	48.8	103	48.1	22	52.4
Febrile neutropenia	15	6.8	10	5.3	5	16.1*	28	10.9	21	9.8	7	16.7*
Anemia	61	27.6	49	25.8	12	38.7	82	32.0	66	30.8	16	38.1
Infection (bacterial/viral)	49	22.2	38	20	11	35.5*	61	23.8	47	22	14	33.3*
pulmonary toxicity	22	10	18	9.5	4	12.9	25	9.8	21	9.8	4	9.5
Cardiovascular complications	5	2.3	2	1.1	3	9.7*	6	2.3	3	1.4	3	7.1*
Neuropathy	13	5.9	11	5.8	2	6.5	18	7.0	15	7	3	7.1
Thrombocytopenia	6	2.7	5	2.6	1	3.2	13	5.1	12	5.6	1	2.4
Hepatic toxicity	6	2.7	5	2.6	1	3.2	6	2.3	5	2.3	1	2.4

*Note*s: Age at initiation of 1L; *Chi‐square test, Fisher exact *p* < 0.05.

AEs tended to be prevalent in patients aged ≥ 60 years. In particular, in the ABVD‐treated subgroup, febrile neutropenia was prevalent in 16.1% of patients aged ≥ 60 years compared to 5.3% in < 60 years old (*p* < 0.05). Likewise, cardiac toxicity was prevalent in 9.7% of patients aged ≥ 60 years compared to 1.1% in < 60 years old (*p* < 0.05).

A majority (89.1%) of ABVD patients received at least one supportive therapy to manage potential AEs induced by cHL treatment, including Granulocyte Colony Stimulating Factor (G‐CSF), Erythroid Stimulating Agent (ESA), blood transfusion, antibiotic/antiviral prophylaxis, and/or other treatments (anti‐emetic, anti‐diarrheal, analgesic, and/or dermatologic, Table 4). The rate of all grade neutropenia 47/58 (81.0%) and febrile neutropenia 8/58 (13.8%) were higher in patients receiving secondary G‐CSF as compared to primary prophylaxis [42/83 (50.6%) and 7/83 (8.4%)] (not presented in the table).

**TABLE 4:** Supportive therapies administered to ABVD‐treated patients, presented by age (< and ≥ 60 years old). AB/AV, antibiotic or antiviral prophylaxis; ESA, Erythroid Stimulating Agent; G‐CSF, Granulocyte colony stimulating factor. *Chi‐square test, Fisher exact *P* < 0.05

	**ABVD (*N* = 221)**						**Overall total (*N* = 256)**					
			**Age < 60**		**Age ≥ 60**				**Age < 60**		**Age ≥ 60**	
	***N* = 221**		***N* = 190**		***N* = 31**		***N* = 256**		***N* = 214**		***N* = 42**	
	***N***	**(%)**	***N***	**(%)**	***N***	**(%)**	***N***	**(%)**	***N***	**(%)**	***N***	**(%)**
**Supportive therapy (at least one)**	197	89.1	168	88	29	93.6	230	89.8	192	89.7	38	90.5
**G‐CSF**	141	63.8	119	62.6	22	71	172	67.2	143	66.8	29	69.1
1° prophylaxis	83	37.6	69	36.3	14	45.2	107	41.8	89	41.6	18	42.9
2° prophylaxis	58	26.2	50	26.3	8	25.8	65	25.4	54	25.2	11	26.2
**ESA**	14	6.3	9	4.7	5	16.1*	15	5.9	9	4.2	6	14.3*
**Blood transfusion**	15	6.8	13	6.8	2	6.5	24	9.4	21	9.8	3	7.1
**AB/AV**	53	24.0	50	26.3*	3	9.7	66	25.8	60	28*	6	14.3

*Notes*: Age at initiation of 1L; AB/AV, antibiotic or antiviral prophylaxis; ESA = erytroid stimulating agent; G‐CSF, granulocyte‐colony stimulating factor. *Chi‐square test, Fisher exact *p* < 0.05.

Death occurred in 19 (7.4%) patients in the overall cohort. Fifteen patients (6.8%) in the ABVD cohort died of causes related to infection (*n* = 7), cHL (*n* = 4), organ failure (*n* = 3), or unknown cause (*n* = 1).

## 4 DISCUSSION

Data collected retrospectively from the charts review of patients diagnosed with cHL over a 4‐year period up to August 2018, showed that ABVD was the most prevalent 1L systemic therapy used across three countries: IL, IT, and SP. High IPS and age ≥ 60 years were indicative of lower PFS both in the overall study population and the ABVD subgroup. Other factors generally associated with poorer prognosis, such as stage IV or the presence of extra‐nodal disease, were also associated with a decrease of 30‐mo PFS in both cohorts, though this difference was not statistically significant.

It has been suggested that PET2 assessments hold a superior prognosis potential to IPS [22, 23, 24]. There continues to be a debate surrounding the effectiveness of early‐response PET assessments to guide de‐escalation of therapy for patients with a high probability of cure after ABVD therapy and escalation for those at higher risk for treatment failure. Stephens et al. have reported unexpected high rates of relapse in PET2‐negative patients treated with six cycles of ABVD [21]. We cannot sufficiently address the prognostic potential of PET2 assessments due to limitations of study design (retrospective chart review). Furthermore, as PET2‐guided treatment strategies have only been incorporated into clinical guidelines in 2018 [25], we may not have adequately captured this information at the time this study was undertaken. Further studies on the utility of PET2 in clinical practice settings would be of value.

The observed rate of PET2‐positive scans (30%) was higher than in clinical trials [14, 18], as often reported in studies in clinical practice settings [26]. This may reflect the higher proportion of stage IV cHL patients included in clinical practice [27]. We observed PET‐adapted strategy of treatment intensity modification occurred in a small fraction of patients. This study reflects patient management in clinical practice and as such, age of patients and higher rates of comorbidity in the clinical practice setting may have influenced the decision to skip treatment escalation. Of note, our retrospective study also included a higher number of stage IV cHL patients (51.2%) than in previous real world studies (28.9% [26]; 11.8% [28]). Furthermore, in the present study, not all patients (approximately 67% of patients receiving ABVD) had an interim PET evaluation at cycle 2; assessments were delayed for approximately 12.6% of patients with PET3 and 13% with PET4.

Nonetheless, persistence of cHL disease, as detected in PET2‐positive scans, was associated with lower survival for ABVD‐treated patients. This observation highlights the pertinence to adapt treatment of unresponsive patients as early as after two cycles of ABVD, and to perform PET2 assessment as per guidelines as suggested in earlier reports [18, 20, 28].

Increasing treatment intensity, most often from ABVD to eBEACOPP, which showed better initial results and is supported by data from several clinical trials [11, 13, 18, 20, 21, 28, 29], exacerbates acute toxicity and long‐term organ damage [20, 28]. High rates of febrile neutropenia are generally attributed to doxorubicin and dacarbazine, while bleomycin also increases the risk of irreversible pulmonary damage [9, 30–32]. Brentuximab vedotin (A) in combination with AVD, an alternative 1L treatment option, is associated with substantially less pulmonary toxicity than ABVD, however myelotoxicity and neurotoxicity are increased (although myelotoxicity can be ameliorated with prophylactic G‐CSF and neurotoxicity is largely reversible). The A+AVD combination appears to be more effective than ABVD for 1L treatment of advanced‐stage cHL [14, 33, 34], and may have a role in treatment of older cHL patients and those with high IPS stage [35, 36], who tend to have less favorable outcomes as shown in this study.

Even in patients with standard of care treatment (ABVD), the use of supportive therapies, especially G‐CSF is extensive. Neutropenia, including febrile neutropenia, was greatly reduced when G‐CSF was used as a primary prophylactic treatment, in line with prior reports [14]. Accordingly, prophylactic G‐CSF was suggested to improve survival outcomes [33]).

In the present study in common clinical practice settings in IL, IT and SP, ABVD was the most prevalent 1L systemic therapy. Survival outcomes remained lower than in clinical trials, especially for older patients and for patients presenting with high IPS. Supportive therapies were used in the majority of patients; reported treatment‐induced toxicities included pulmonary and cardiac toxicities and febrile neutropenia. Evidence for use of PET scan assessments and consequently, PET‐guided treatment modification, was limited during the study period. Unmet needs continue to exist with respect to the balance between therapeutic efficacy and safety risks of 1L therapy.

## 5 STATEMENT OF SIGNIFICANCE

Patients with higher IPS consistently presented with poorer survival outcomes. Most of the patients were treated with ABVD as 1L chemotherapy across all centers in three countries.

Despite being recommended after two cycles of 1L chemotherapy in advanced cHL treatment guidelines, evidence for use of PET scan assessments and consequently, PET‐guided treatment modification, was limited in common clinical practice in the three included countries during the study period.

There remains an unmet need for both safer and more efficient 1L therapy for advanced cHL patients presenting with unfavorable prognostic factors and low therapeutic response after two cycles of 1L chemotherapy.

## FUNDING

Funding for this research was provided by Takeda Pharmaceuticals International AG.

## CONFLICT OF INTEREST

The authors declare that there is no conflict of interest that could be perceived as prejudicing the impartiality of the research reported.

## ETHICAL APPROVAL

This observational study was conducted in accordance with the Declaration of Helsinki, other legal and regulatory requirements, as well as with scientific purpose, value and rigor and following generally accepted research practices described in Good Pharmacoepidemiology Practices (GPP) guidelines issued by the International Society for Pharmacoepidemiology (ISPE). All data were handled in strictest confidence in conformity with national and international data protection regulations (such as Directive 95/46/EC).

Good Epidemiological Practice (GEP) recommends the study design and data protection aspects be approved by an EC, and, for publication in peer‐reviewed journals or other purposes, approval of an EC is recommended. However, some countries only require EC submission for interventional research only. Where required, the study protocol was reviewed and approved by a duly constituted independent ethics committee at a local level before initiation of data collection.

## CONSENT TO PARTICIPATE

Respondents were informed about confidentiality in the statement of informed consent.

## CONSENT TO PUBLICATION

Not applicable.

## AUTHORS’ CONTRIBUTIONS

AA has provided consultancy and received Honoraria from Takeda, Gilead, Pfizer; has received Research funding from Janssen, BMS. GG has provided consultancy for Takeda, Janseen, Kyowa Kyrin, Gentili; has served on Advisory Board of Janseen, Celgene, Roche, Takeda, Kite, Kyowa Kyrin; has received Financial research support Celgene. FT, FG, NBE, MD, AZ are employees of Takeda. SGB is employee of Kantar Health Division France. No other potential conflict of interest relevant to this article is reported.

## Data Availability

The datasets generated during and/or analyzed during the current study are available from the corresponding author on reasonable request.
